# Nonorthogonal Aerial Optoelectronic Platform Based on Triaxial and Control Method Designed for Image Sensors

**DOI:** 10.3390/s20010010

**Published:** 2019-12-18

**Authors:** Quanchao Li, Shuyan Xu, Yulei Xu, Lei Li, Liu Zhang

**Affiliations:** 1 Key Laboratory of Airborne Optical Imaging and Measurement, Changchun Institute of Optics, Fine Mechanics and Physics, Chinese Academy of Sciences, Changchun 130033, China; xusy@ciomp.ac.cn (S.X.); yuleixu@ciomp.ac.cn (Y.X.); lilei_heu@hotmail.com (L.L.); 2 University of Chinese Academy of Sciences, Beijing 100049, China; 3 College of Instrumentation and Electrical Engineering, Jilin University, Changchun 130033, China; zhangliu@jlu.edu.cn

**Keywords:** aerial optoelectronic platform, nonorthogonal, image sensor, tracking, bandwidth, stability accuracy

## Abstract

A traditional aerial optoelectronic platform consists of inside and outside multilayer gimbals, while an internal gimbal and drive components occupy the internal space where optical sensors are located. In order to improve the replaceability of optical sensors and to increase their available space, this paper introduces a nonorthogonal aerial optoelectronic platform based on three axes; we carried out research on its drive control method. A three-dimensional structure of an aerial optoelectronic platform was designed. A noncontact drive of a linear voice coil motor was introduced, and a drive control scheme of a proportional integral and a disturbance observer was adopted. Finally, simulations and experiments were carried out. Results showed that the aerial optoelectronic platform could effectively release three times the image sensor space, and the servo bandwidth was 60.2 Hz, which was much better than that of traditional two-axis and four-gimbal platforms. The stability accuracy of the system reached 4.9958 micron rad, which was obviously better than that of traditional gimbals. This paper provides a reference for the design of new optoelectronic platforms.

## 1. Introduction

An aerial optoelectronic platform can continuously measure its attitude and position [1,2] due to carrier disturbance (e.g., missiles, aircraft, and ships), accurately maintain a dynamic attitude reference, and realize the automatic tracking [3,4,5] of a maneuvering target through image sensors. In recent years, it has been widely used in military and in civil fields such as public security, fire protection, and environmental monitoring [6,7]. Depending on the carrier platform, an aerial optoelectronic platform is divided into fixed-wing combat-aircraft, airborne-helicopter, and unmanned-airborne optoelectronic platforms. The solution for acquiring images by linear array charged coupled device (CCD) plus scanning motion is still widely used, especially in cases that require a large field of view and high image resolution. However, high resolution alone cannot guarantee high image recognition accuracy. Therefore, image recognition must fully take advantage of high resolution, and at the same time overcome the effect of scanning motion from the algorithm, so that an error in mechanical transmission will not directly affect the final image recognition accuracy.

Although the optical sensor has a lower detection range and coverage and has higher visibility requirements than the radar, the biggest advantage of the optical sensor is that it has a shooting function. It can be photographed and enlarged for careful observation. It is very intuitive and the target is more accurate than the radar. Moreover, the optical equipment is not afraid of interference. Even if the local area releases electromagnetic interference, it can ensure sufficient detection results. At the same time, the optical sensor can also be used for mapping, and can learn the development of natural disasters such as earthquakes, volcanic eruptions, and fires in time. With manned or unmanned aerial vehicles, it is highly effective, accurate, and has a wide detection range.

With the improvement of carrier-platform performance, the development of aerial photoelectric sensors should have characteristics of precision, miniaturization, intelligence, and multitasking integration [8]. With the development of optoelectronic sighting equipment towards all-weather, multispectral, and multimode sensors, the image processing technology of photoelectric sensors has also developed from a single target capture and tracking function to intelligent, multi-mode fusion, quantitative analysis, etc., which has increased the difficulty of acquiring high-quality images, resulting in the platform’s increasing demands. What these characteristics have in common is that they require many types of photoelectric sensors, high performance, a small system size, and panoramic perception. For example, Israel’s TOPLITE company has a two-axis four-stabilized gimbal equipped with multiple optical sensors, such as visible light cameras, infrared cameras, and laser rangefinders with a stable accuracy of 25 microrad [9]. Lockheed Martin’s AM/AAQ-30 Targeting System (TSS) for the U.S. Marines’ AH-1Z Cobra Attack Helicopter is the most advanced multi-sensor photoelectric fire control system in the world. TSS has large-aperture, medium-wave, forward-looking infrared sensors, high-resolution color-television cameras, laser target indicators, human-eye-safe laser rangefinders, laser spot trackers, and inertial measurement devices, with a stable accuracy of 20 microrad [10]. Developed by Canada’s L-3 communications company’s WESCAM branch, MX-15 is a medium-sized sensor turret with a weight of 42.7 kg [8]. It can carry six kinds of photoelectric sensors at the same time.

**Figure 1:**
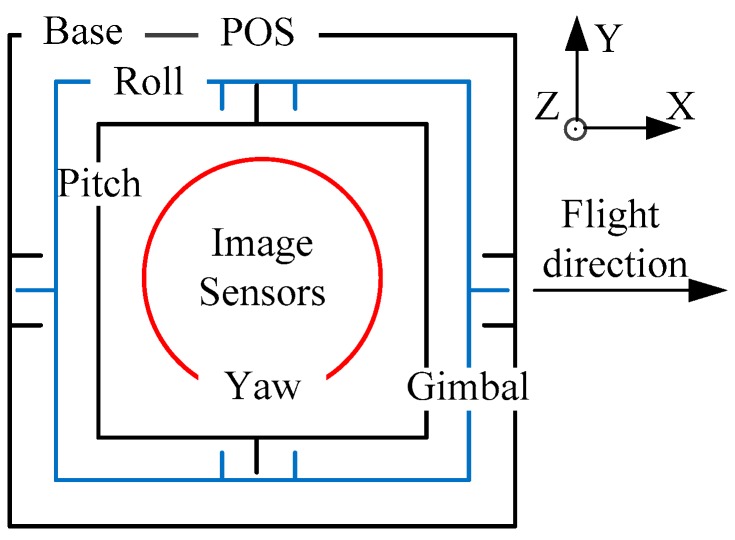
Traditional aerial-optoelectronic-platform structure.

A traditional aerial optoelectronic platform consists of a multilayer gimbal inside and outside [7]. As shown in Figure 1, the image sensor is surrounded by an annular gimbal of orthogonal axes, and the gimbal and drive components occupy a large part of the space. As the number of multi-image sensor tasks (such as infrared cameras, visible light cameras, and laser ranging) and multi-axis [11,12] motion requirements increases, the size of multilayer gimbals also increases, resulting in poor system stiffness. The shape of the inner gimbal is usually designed according to the shape of the image sensor, which encounters obstacles when it is necessary to replace image sensors for different uses and types of performance, resulting in low platform reusability.

A traditional aerial optoelectronic platform is generally driven by a torque motor [13]. The drive motor is directly connected to the inner gimbal through the shaft assembly, and the image sensor is driven by the inner gimbal. The frictional disturbance of the motor and the shaft system is directly transmitted to the image sensor through the shaft system, which reduces image-acquisition accuracy. In addition, the gimbal shape is designed as a ring structure due to the driving form. However, a large ring structure has low fundamental frequency and a poor rigidity. When the motor is loaded with the torque to the ring structure for stability control, system bandwidth and other types of performance are severely limited.

In order to solve the problems of a large occupied gimbal and low image-sensor replacement in the existing aerial optoelectronic platform of an orthogonal axis system, a nonorthogonal aerial triaxial-based optoelectronic platform was designed that is mainly used in unmanned aerial vehicles, and a new linear voice coil motor drive is introduced in this paper. High-precision control technology of a proportional integral and disturbance observer is introduced in detail, and parameters such as system bandwidth and stability accuracy were tested and verified.

## 2. Aerial Optoelectronic Platform Design

### 2.1. Advantages of Three-Axis Compensation

**Figure 2:**
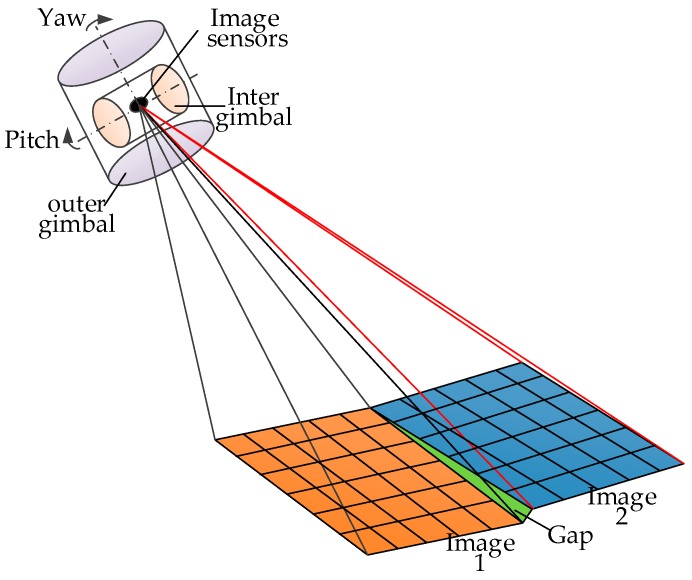
Two-axis compensation image stitching gap.

Traditional aerial optoelectronic platforms generally use two-axis [14] motion, that is, yaw- and pitch-direction motion to compensate for the image sensor, but there are certain problems with this. The maximum disturbance of the optoelectronic platform on the carrier comes from perturbation in the roll direction. The yaw direction is affected by the airflow, and motion in the pitch direction changes relatively slowly. When the carrier is performing a large elevation movement or high-speed maneuvering, the overlap ratio of the image has a greater relationship with the angle of view of the image sensor. This is because image-sensor focal length is long and field of view is small. When certain interference is applied from the outside, it is difficult for two-axis compensation to uniformly respond to the carrier movement, and multiple obtained images have a slit when stitched, as shown in Figure 2. In particular, in the case of a long focal length and small field of view, the accuracy requirements of the system for each shafting are increased, and sensitivity to disturbance is enhanced.

In addition, the three-axis motion of the carrier introduces three axes of disturbance, there is a lot of coupling between them, and the compensation of the two axes leads to information loss. In the past, image sensors used a tracking mode, but in the case of cloud occlusion at a distance, much of the information was blocked. In particular, the longer the focal length was, then the more information there was was contained in the squint layer, and the more disturbances there were, the more difficult it was for the image sensor to track the target. However, if the optoelectronic platform adopted a stepping imaging mode, that is, taking multiple photos at different angles to obtain complete information of the target, then it would no longer be necessary to continuously track the target, thereby reducing the difficulty of target acquisition. In summary, there are many application limitations in two-axis compensation. For this reason, this paper studies the design of a three-axis compensation mechanism.

### 2.2. Nonorthogonal Triaxial Mechanism

**Figure 3:**
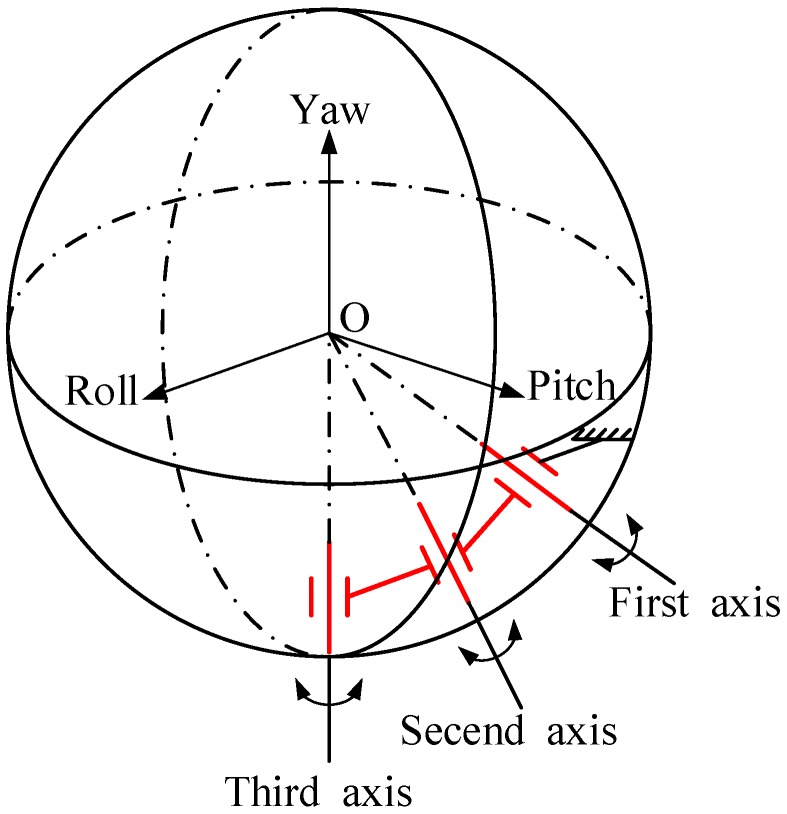
Nonorthogonal three-axis motion mechanism.

In order to reduce the space occupied by multilayer gimbals and the direct drive motor, a nonorthogonal three-axis motion mechanism was studied, as shown in Figure 3. The motion module of the optoelectronic platform is made up of three nonorthogonal shafts connected in a series. The virtual extension lines of the three axes intersect at the same point, and this is exactly the spherical center of the space. Design considerations were as follows: first, the space of the image sensor could be released to the utmost; and second, the three axes intersect at the same point in space, which maximizes average torque and facilitates servo control. During use, we tried to ensure that the center of mass for the image sensor was located near the origin of the space sphere coordinates. The three rotating shafts could perform a certain angular rotational motion about the axis, wherein the third virtual rotating shaft coincided with the vertical axis of the passing center in the design, and the coupling of the three axial angular motions realized the displacement movement of the image sensor.

The direction of the third axis pointed vertically to the center of the sphere; the angle between the second and the third axis was 21°, and the angle between the first and the second axis was 23°. The transmission part of this platform is similar to the joint part of a robotic arm. Due to this, the D–H modeling method proposed by Denavit and Hartenberg [15,16] was used for kinematic modeling. This method is mainly used in robot kinematics. It establishes a coordinate system on each rod and realizes coordinate transformation on two links through homogeneous coordinate transformation. In a multilink series system, by using the homogeneous coordinate transformation multiple times, the relationship between the first and last coordinate system can be established.

**Figure 4:**
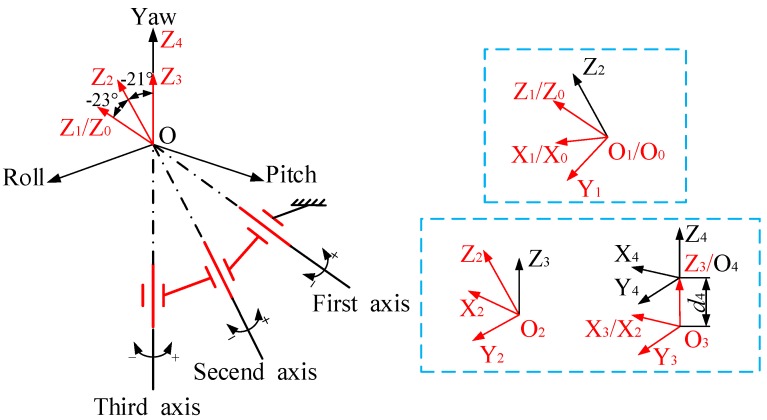
Establishment of coordinate system for each platform rotation axis.

According to the D–H modeling method, the coordinate system of each rotation axis of the platform was established, where the *Z*-axis was the direction of each rotation axis, and the three axes intersected at a certain point in space; this point is the coordinate origin of each coordinate system. The *X*-axis direction was determined by the normal direction of the intersecting axis, and the *Y*-axis direction was determined according to the right-hand rule. Finally, the coordinate system of each rotation axis was established, as shown in Figure 4. The specific coordinate-system establishment process is not described again.

After establishing the coordinate system of each axis, the D–H parameters needed to be determined. The D–H parameters are shown in Table 1, where *θ_i_* is the rotation angle around the *Z*-axis, *d_i_* is the distance between the adjacent common perpendicular on the *Z*-axis, *a_i_* is the length of each male perpendicular line, and *α_i_* is the angle between adjacent *Z*-axes. Only *θ* (0) is variable, where 0 represents the initial state so that the homogeneous coordinate transformation of the {0} system to the {4} system could be obtained, such as in Formulas (1) and (2). The homogeneous coordinate transformation formula of the platform could be obtained by bringing the data in the parameter table into the two formulas.

(1) $${}_{0}^{4}T={}_{0}^{1}T{}_{1}^{2}T{}_{2}^{3}T{}_{3}^{4}T$$

(2) $${}_{i-1}^{i}T=\left[\begin{array}{cccc} \cos\theta_{i} & -\sin\theta_{i} & 0 & a_{i-1}\\ \sin\theta_{i}\cos\alpha_{\left(i-1\right)} & \cos\theta_{i}\cos\alpha_{\left(i-1\right)} & -\sin\alpha_{\left(i-1\right)} & -\sin\alpha_{\left(i-1\right)}d_{i}\\ \sin\theta_{i}\sin\alpha_{\left(i-1\right)} & \cos\theta_{i}\sin\alpha_{\left(i-1\right)} & \cos\alpha_{\left(i-1\right)} & \cos\alpha_{\left(i-1\right)}d\\ 0 & 0 & 0 & 1 \end{array}\right]$$

**Figure 5:**
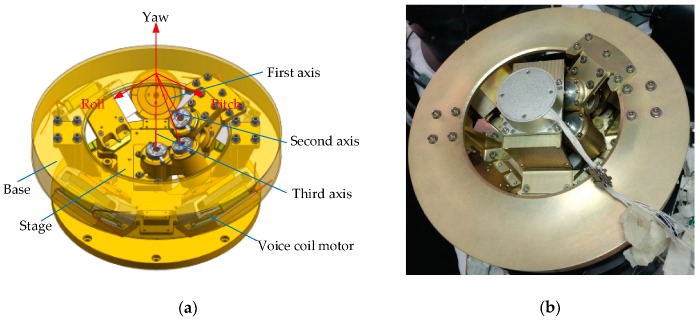
Photoelectric platform three-dimensional map: (**a**) 3D model diagram; (**b**) physical map.

The driving part mainly includes a joint-like mechanism, an upper and lower support gimbal, a base, a stage, four voice coil linear motors, angle sensors, and fiber optic gyroscopes. The image sensors can be connected to the stage in an appropriate manner according to specific needs. The intersection of the orthogonal roll and the pitch and the yaw axis of the optoelectronic platform coincide with the intersection of the three virtual axes of the joint mechanism, which can realize the movement requirements of the roll, pitch, and yaw angles of the image sensor. Figure 5 is a three-dimensional-model and a physical diagram of the designed nonorthogonal three-axis optoelectronic platform with a diameter of 300 mm and a height of 100 mm.

In the design of the platform, a linear voice coil motor drive was introduced. The four linear voice coil motors were symmetrically arranged in the three-axis intersection, and the center position of the motor points was vertical to the three-axis intersection. The coil portions of the four motors were respectively fixed to the base, and the permanent magnet portion of the motor was fixed to the stage. The lower end of the stage was equipped with a nonmetallic material retaining ring for the limit function of the roll and pitch angles, and the yaw angle limit post was mounted onto the base.

### 2.3. Drive Program Considerations

#### 2.3.1. Voice-Coil-Motor Principle

The linear voice coil motor is a linear motor that was designed on the basis of the Lorentz force principle. It converts electrical energy into mechanical energy and realizes linear motion, which can eliminate the adverse effect of the intermediate transmission link on system performance. The regular interaction of magnetic poles between the magnetic field of the magnetic steel and the magnetic field generated by the energized coil conductor produces a regular motion [17,18].

Because the voice coil motor is a noncommutated power device, its positioning accuracy and force control [19,20] are completely dependent on the feedback and control system. Accuracy is mainly determined by the controller, and the influence of the voice coil motor is relatively small. With proper positioning feedback and a sensing device, positioning accuracy and acceleration can achieve the desired effect.

The magnitude of the force generated by the linear voice coil motor depends on design structure and current intensity: F = B × L × I, where B represents the magnetic induction strength, L represents the coil length, and I represents the current magnitude. The relationship between the current and the generated force is expressed as torque sensitivity Kf in the use of a linear voice coil motor. The unit of Kf was defined as N·m/A in the design.

The coil winding was placed in a uniform air-gap magnetic field, and the winding direction was perpendicular to the direction of the uniform magnetic field. When the current flowed through the winding, ampere force was generated to drive the load to reciprocate. By changing the strength and direction of the current, amperage magnitude and direction can also be changed.

#### 2.3.2. Voice-Coil-Motor Design and Analysis

**Figure 6:**
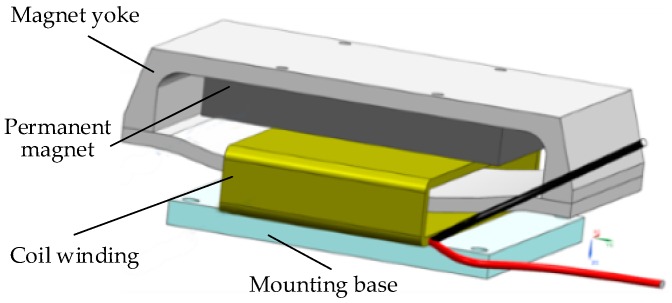
Voice-coil-motor composition.

The structure and size of the voice coil motor were designed, which included permanent magnets, coils, and yokes, as shown in Figure 6. After the preliminary design, electromagnetic simulation software was used for verification, and the shape and related dimensions of the motor were modified according to the results. After repeated iterations, simulation results reached the requirements.

The NdFeB materials have excellent physical properties and low cost, which is why they are widely used in applications with high standards on motor weight and performance. In this paper, NdFeB was used as the permanent magnet material of the motor. The yoke was made of electromagnetic pure iron DT4C with high magnetic permeability and no residual magnetism. The coil was made of self-adhesive enameled wire. Therefore, the wound coil itself had sufficient mechanical strength and could be directly fixed to the mount by adhesive. No additional gimbal was required, and the weight of the moving part could be reduced.

**Figure 7:**
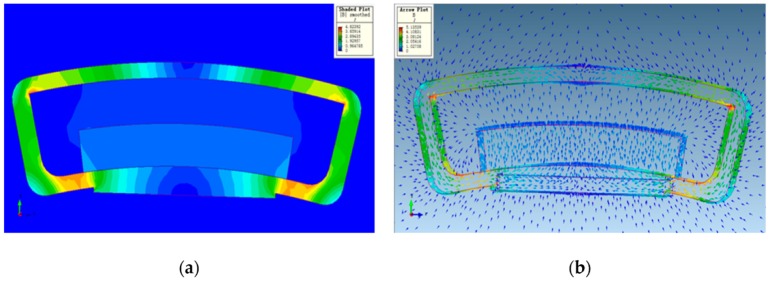
Electromagnetic simulation analysis results of voice coil motor: (**a**) magnetic induction distribution; (**b**) magnetic line distribution.

**Figure 8:**
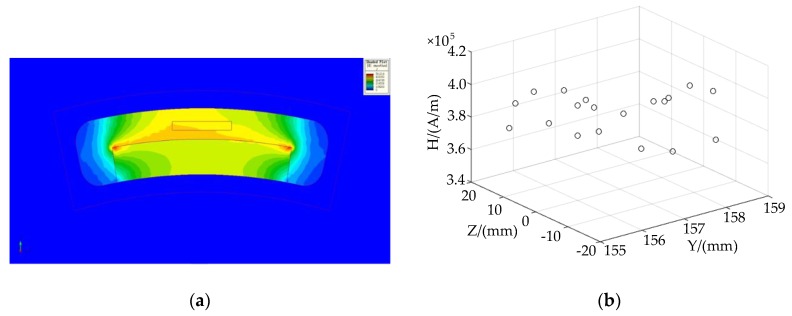
Analog value of magnetic field strength H: (**a**) magnetic field strength H test area; (**b**) magnetic field strength H distribution.

The magnitude of the ampere force applied to the coil was proportional to the strength of the magnetic field. Therefore, in order to obtain stable thrust and moment, a uniform magnetic field with as small of a change in the magnetic-field strength as possible should be generated within the stroke range of the coil. It can be seen from the simulation results of the voice coil motor in Figure 7 that magnetic-field strength changed little at the air gap of the permanent magnet that was close to the central position to the yoke, and the driving force generated by the coil was uniform. Therefore, the motion of the image sensor should be controlled as closely as possible to the central area. We selected the center area of the two magnetic poles and randomly chose the 19 points shown in Table 2. The measured magnetic-field-strength regions and distribution trends are shown in Figure 8. The magnetic field strength H was distributed around 3.9 × 10^5^ A/m, which was relatively stable.

**Figure 9:**
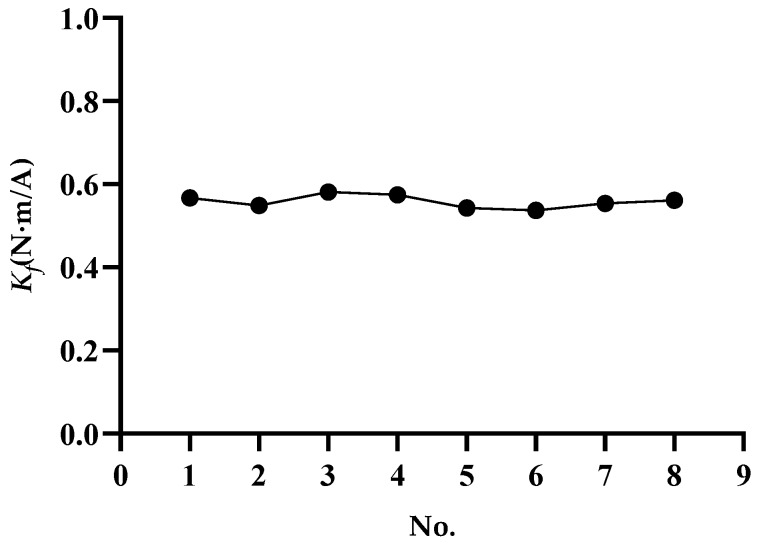
Torque-sensitivity repeatability test.

According to the design and simulation results, a linear voice coil motor was produced. Peak voltage of the motor was 30.5 V, peak current was 3.6 A, and torque sensitivity Kf was 0.55 N·m/A. Figure 9 shows the torque-sensitivity values for eight measurements, indicating that the voice coil motor had good repeatability. The peak torque of the motor was 2 N·m, the continuous blocking torque was 0.89 N·m, the range of motion was 6°, and the total weight was 560 g.

#### 2.3.3. Voice-Coil-Motor Drive-Control Strategy

**Figure 10:**
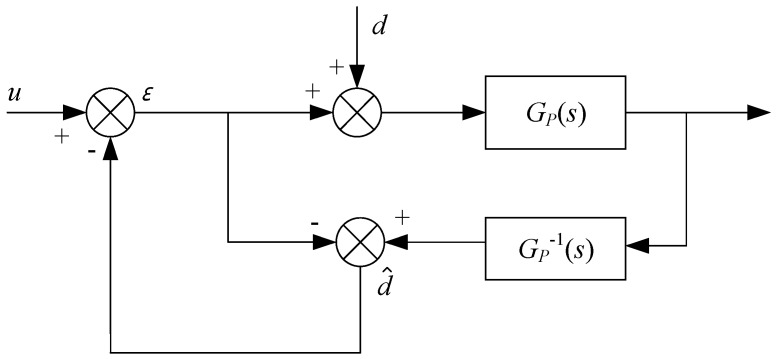
Disturbance observer.

Since simple proportion integration (PI) control has a weak anti-interference ability, an interference observer was added to enhance the anti-interference of the voice-coil-motor control. The basic idea of the Disturbance Observer (DOB) is to input the difference between the actual object and nominal model output caused by external torque disturbance and the change of model parameters equivalent to the control terminal [21,22,23], that is, equivalent interference was observed, and an equal amount of compensation was introduced in the control to achieve complete interference suppression. The basic idea is shown in Figure 10.

*G_P_*(*s*) is the transfer function for the object, *d* is the equivalent interference, $\hat{d}$ is the observed interference, and *u* is control input. From Figure 10, the estimated value $\hat{d}$ of the equivalent interference is:

(3) $$\begin{array}{cc} \hat{d} & =(\varepsilon +d)\cdot G_{P}(s)\cdot G_{P}{}^{-1}(s)-\varepsilon \\ & =d \end{array}$$

The above formula shows that the above method can calculate accurate interference estimation and compensation. For an actual physical system, interference-observer (Figure 10) implementation has the following problems:

First, under normal circumstances, the relative order of *G_P_*(*s*) is not 0, and its inverse is physically impossible; second, the exact mathematical model of object *G_P_*(*s*) is not available; third, considering the influence of measurement noise, the control performance of the above method would decrease.

**Figure 11:**
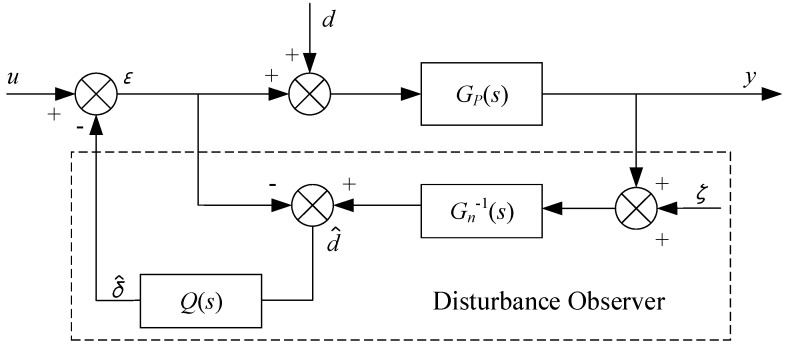
Block diagram of disturbance observer.

A natural idea to solve the above problem is to string a low-pass filter *Q*(*s*) behind $\hat{d}$, and to replace *G_P_*^−1^(*s*) with the inverse, *G_n_*^−1^(*s*), of the nominal model *G_n_*(*s*); the block diagram where the interference observer is inside the dotted line is shown in Figure 11.

In the figure above, ζ is measurement noise, and *u*, *d*, and ζ are the input. By the superposition principle, system output y is:

(4) $$y=G_{UY}(s)u+G_{DY}(s)d+G_{\xi Y}(s)\xi$$

The following solutions were obtained:

(5) $$G_{UY}(s)=\frac{G_{P}(s)G_{n}(s)}{G_{n}(s)+[G_{P}(s)-G_{n}(s)]Q(s)}$$

(6) $$G_{DY}(s)=\frac{G_{P}(s)G_{n}(s)[1-Q(s)]}{G_{n}(s)+[G_{P}(s)-G_{n}(s)]Q(s)}$$

(7) $$G_{\xi Y}(s)=\frac{G_{P}(s)Q(s)}{G_{n}(s)+[G_{P}(s)-G_{n}(s)]Q(s)}$$

It can be seen from the four equations above that *Q*(*s*) is a very important link in the design of the disturbance observer. Firstly, in order to make the *Q*(*s*)*G_n_*^−1^(*s*) regular, the relative order of *Q*(*s*) should be no less than the relative order of *G_n_*(*s*); second, the bandwidth design of *Q*(*s*) is a compromise between robust stability r and the interference rejection capability of the interference observer.

Suppose *Q*(*s*) is the ideal low-pass filter, that is, in the low-frequency band, when *f* ≤ *f*_0_, *Q*(*s*) = 1, and in the high-frequency band, when *f* ≥ *f*_0_, *Q*(*s*) = 0. At low frequencies, there is:

(8) $$G_{UY}(s)=G_{n}(s),\;G_{DY}(s)=0,\;G_{\xi Y}(s)=-1$$

The above equation shows that, in the low-frequency band, even if *G_P_*(*s*) ≠ *G_n_*(*s*) or there is uncertainty, then the interference observer can still make the response of the actual object consistent with the response of the nominal model, namely, the controller has a certain robustness to object parameter variation. *G_DY_*(*s*) = 0 indicates that the interference observer completely suppresses low-frequency interference in the *Q*(*s*) band. *G_ζY_*(*s*) = −1 indicates that the disturbance observer is very sensitive to low-frequency measurement noise. Therefore, in practical applications, appropriate measures must be considered to reduce low-frequency noise in motion-state measurements.

In the high-frequency band, where *Q*(*s*) = 0, there is

(9) $$G_{UY}(s)=G_{P}(s),\;G_{DY}(s)=G_{P}(s),\;G_{\xi Y}(s)=0$$

The above equation shows that, at high frequencies, the disturbance observer is not sensitive to the measurement noise, but it does not have an inhibitory effect on the perturbation of the object parameters and external disturbances.

## 3. Test and Results

The demonstration system was built to verify the drive and high-precision control technology of the three-axis nonorthogonal aerial optoelectronic platform. We fixed the optoelectronic platform onto the test turret, and connected the platform and power supply, monitor, and emulator to demonstrate the parameters of the platform.

**Figure 12:**
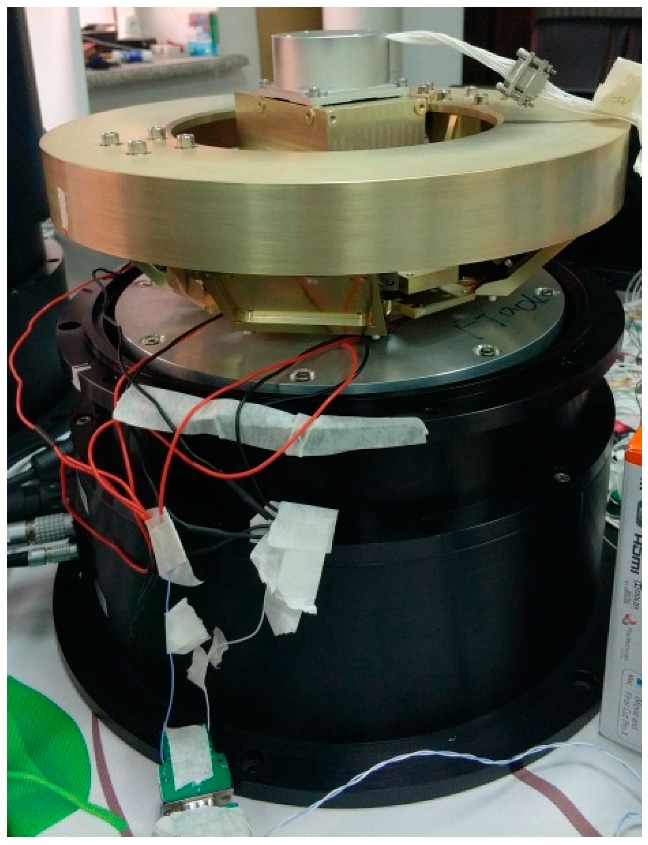
Three-axis nonorthogonal-optoelectronic-platform demonstration device.

Taking the yaw axis as an example, the inner carrier of the rotating platform is controlled by the voice coil motor to check angular-motion range, angular velocity, and angular-acceleration capabilities; the servo control bandwidth of the optoelectronic platform was tested. By commanding the turntable to make a sinusoidal motion according to the set value, we collected the gyro feedback characteristic curve on the load connection board, verified the stability accuracy of the photoelectric platform, and verified the stability of the optoelectronic platform. The physical diagram of the system demonstration device is shown in Figure 12.

### 3.1. Motion-Range Test

**Figure 13:**
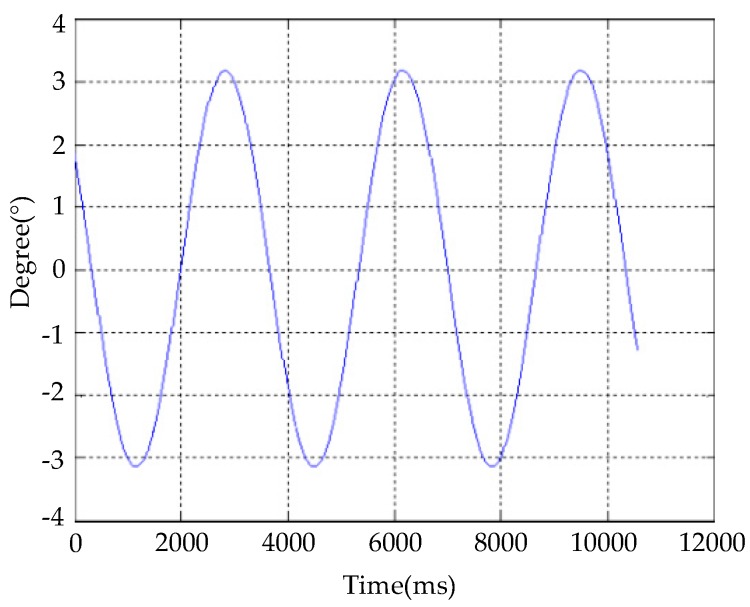
Angle-range test.

The optoelectronic platform was fixed on the analog turntable, and the joint shaft was driven by driving the voice coil motor. The end of the joint shaft followed the shaft system to perform the corresponding motion, and the angular-motion range was checked; test results are shown in Figure 13. The stage had a range of motion of ±3.2° with respect to the base, thereby meeting system specifications. Since the structure limits the movement angle of the platform to a certain extent, in the actual design, the target parameter range of the image sensors should be fully considered, and the angular relationship of the shaft system should be reasonably set.

### 3.2. Stability-Accuracy Test

**Figure 14:**
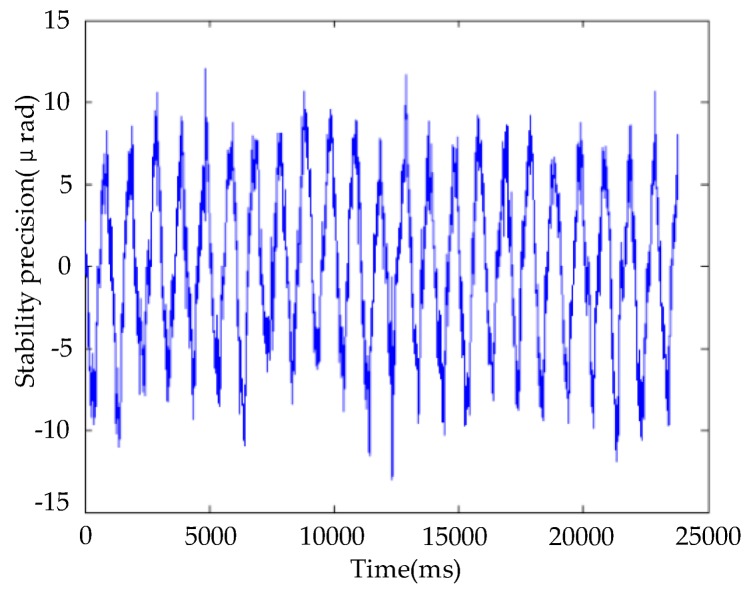
Stability-accuracy test.

The analog turntable was turned on and it was controlled to perform a sinusoidal motion according to 1° amplitude and 1 Hz frequency. At the same time, the feedback characteristic curve of the fiber-optic gyroscope, fixedly connected with the stage, was collected, as shown in Figure 14. Test results showed that the root-mean-square value of the disturbance error was 4.9958 microrad.

### 3.3. System-Bandwidth Ttest

**Figure 15:**
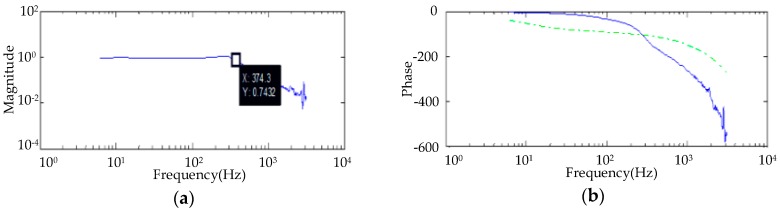
Stable bandwidth test: (**a**) actual model bandwidth; (**b**) fitting model bandwidth.

Pseudorandom noise disturbance was applied to the stage in the photoelectric platform, and a closed-loop servo control bandwidth test of the photoelectric platform was carried out; test results are shown in Figure 15. The stable servo control bandwidth was 60.2 Hz, which is far superior to that of traditional two-axis four-gimbal structures.

### 3.4. Motion-Speed Test

**Figure 16:**
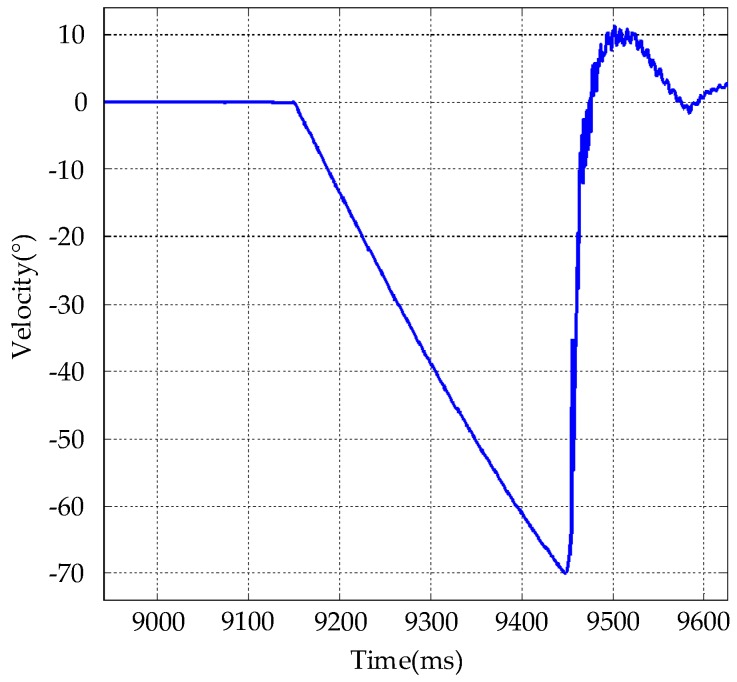
Maximum speed and acceleration test.

A traditional torque motor has poor speed-response characteristics, so it was necessary to measure the speed of the system. Taking the yaw axis as an example, speed input with an angular velocity of no less than 60°/s and an angular acceleration of no less than 200°/s^2^ was used for the step test, and the gyro feedback step characteristic curve was acquired; test results are shown in Figure 16. The measured angular velocity could reach 70°/s, and we also calculated the angular acceleration of the corresponding segment, which could reach 458°/s^2^.

**Figure 17:**
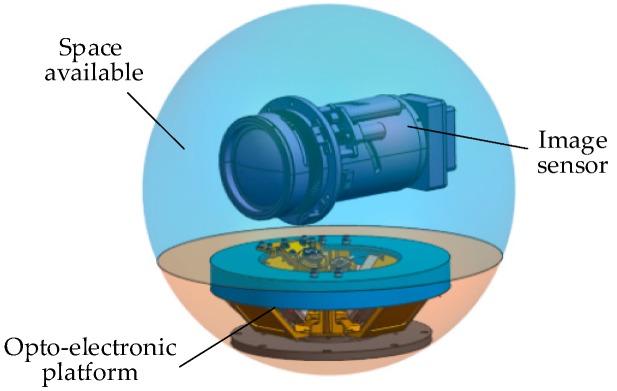
Schematic diagram of payload ratio.

The proportion of the optoelectronic platform in the whole sphere space is shown in Figure 17. The joint drive mechanism only occupies part of the spherical crown space, and the center space of the sphere can be used to accommodate the image sensors, circuit boards, and other related devices. By calculating the volume comparison of the two parts, we found that the payload volume ratio of our aero-optical platform was 21,855,677.58:6,874,290.3 mm^3^ ≈ 3.18:1.

## 4. Discussion

### 4.1. Platform Cost Considerations

The difference in structural form was largest between the driving form and the gimbal structure. Compared with torque motors, the manufacturing process of voice coil linear motors is relatively simple. It could obtain larger torque output under limited volume and weight, it has good dynamic response characteristics, and it is cheaper. In addition, the number of supports for traditional platform structures is relatively large, and some parts are difficult to process. For example, more important support structure collimators, which are commonly used, cannot be produced by casting in small batches, or even in single-piece production processes, while material removal can only be done by machining. Many places that cannot be processed by tools can only be removed by means of material processing, such as an electric spark. On the one hand, the processing cost is significantly increased, and on the other hand, the EDM process cycle is long, which greatly increases the production cycle of the equipment. Compared with a traditional aviation optoelectronic platform, this open structure makes it easier to replace different image sensors to obtain the desired image according to the actual situation, and the platform has a high utilization rate. In summary, the new platform has obvious cost and cycle advantages over traditional platforms.

### 4.2. Platform Performance Analysis

Experiment results were verified for multiple indicators of the platform. The angular velocity of the platform could reach 7°/s and acceleration could reach 458°/s^2^, which indicates that the designed voice coil linear motor has a good dynamic performance, and the motor performance directly affects servo bandwidth capability. The measured range of the movement angle of the platform was ±3.2°. Although the movement angle of the designed platform can be adjusted according to structural changes, the structure needs to be changed, which increases the cost and cycle. It is not as flexible as a traditional platform with large angles, which results in certain limitations in wide-FOV (field of view) search applications.

The servo bandwidth was 60.2 Hz, which is significantly improved compared to a traditional optoelectronic platform. High bandwidth enables the platform to quickly respond to recapture targets when encountering external interference, such as sudden high-angle maneuvering of the aircraft or wind disturbance that causes the target to exceed the sensor detection area, which can effectively reduce image information. High bandwidth is a prerequisite for high system stability and accuracy. The main problem in improving stability accuracy is that the system bandwidth is not high. In addition to the bandwidth of the servo drive, bandwidth is also closely related to system structural stiffness, friction, and the drive method. Stability accuracy was 4.9958 microrad, which is obviously improved compared to a traditional platform. High stability accuracy guarantees high-quality image acquisition, and it is also the most important link for optical sensors to accurately capture targets and continuously track them.

At present, traditional platforms are limited by structure and driving characteristics, so it is difficult to obtain high stability accuracy, and the disadvantages are obvious in practical applications. For example, for stable video, image stabilization is necessary. There are three main methods of image stabilization: optical, mechanical, and electronic. Optical image stabilization is an optical sensor that adaptively adjusts the optical path to stabilize an image. Electronic image stabilization uses electronic algorithms to perform motion filtering and compensation on each video to obtain a stable image, but with the drawback of the problem of field depth. Because the target in the scene is in a large depth-of-field range, the relative position and size of each target in the image sequence changes with the movement of the camera. Targets with different depths of field have different motion vectors. It is impossible to stabilize both near and far vision with one level of compensation. Mechanical image stabilization uses a gyro sensor and other devices to detect the shake of the camera platform, and then adjusts the servo system to stabilize the image. High stability accuracy means that the servo system has a good suppression effect on platform shake to obtain stable video.

In addition, due to the use of an open structure, compared to the two-axis four-gimbal structure, this design can effectively increase the load-volume ratio, as well as the size and number of installed image sensors, and can simultaneously replace the actual image sensors as needed. The replacement rate of optical sensors has, therefore, been improved. In summary, the main parameters of the designed platform and traditional platforms of the same level are compared in Table 3. In addition to the limited movement angle, the bandwidth, stability accuracy, and load-volume ratio were significantly improved.

## 5. Conclusions

We proposed a nonorthogonal three-axis aviation optoelectronic platform that has a new structure and driving method to improve the load-volume ratio and stability accuracy. The kinematics of the nonorthogonal three-axis mechanism were studied. The structure adopted an open-space layout, which improved the load plot ratio. The introduction of a voice coil motor drive reduced the transmission of frictional disturbance in direct drive motors, and the use of proportional control combined with a disturbance-observer drive control scheme effectively improved system performance. The platform servo bandwidth was 60.2 Hz, which can quickly respond to external interference and reduce the lack of image information. Stability accuracy was 4.9958 microrad, which guarantees that the image sensor can obtain high-quality video-image information, and the target tracking was more stable. The load-volume ratio was 3.18:1, which means that the platform can be loaded with more image sensors to meet multitasking and multiscenario task requirements. Although the motion angle restricts the application of a wide field of view, task requirements can be met by replacing large field image sensors.

## Figures and Tables

**Table 1:** Denavit and Hartenberg (D–H) parameters.

*i*	*a*_(*i*−1)_	α_(*i*−1)_	*d_i_*	*θ_i_*
1	0	0	0	*θ*_1_ (0)
2	0	−23°	0	*θ*_2_ (0)
3	0	−21°	0	*θ*_3_ (0)
4	0	0	*d*_4_	0

**Table 2:** Random magnetic field strength H test.

X/(mm)	Y/(mm)	Z/(mm)	Value/(A/m)
0	155.409	−14.2893	399,684
0	155.47	−9.42965	414,104
0	155.278	−5.11711	417,289
0	155.086	1.77009	411,519
0	155.214	9.04349	396,813
0	155.597	15.9307	372,647
0	157.195	15.5445	357,277
0	157.131	9.75152	380,283
0	157.322	−9.68711	400,714
0	157.131	−14.8364	375,973
0	158.217	−13.9997	374,636
0	158.6	−8.20669	396,512
0	158.664	−0.160898	391,982
0	158.728	7.3056	377,198
0	158.089	12.9698	366,686
0	156.428	14.5146	371,012
0	156.747	9.68715	384,173
0	157.067	−9.62275	402,452
0	156.364	−14.9652	383,107

**Table 3:** Comparison of main index parameters between our platform and traditional platforms.

Optoelectronic Platform	Nonorthogonal Triaxial (20 kg)	Two-Axis Four-Gimbal (20 kg)
Servo bandwidth	60.2 Hz	30 Hz
Stability accuracy	4.9958 microrad	25+ microrad
Load-volume ratio	3.181	1:2.5

## References

[1] Zhang Y.S., Yang T., Li C.Y., Liu S.S., Du C.C., Li M., Sun H.L. (2014). Fuzzy-PID control for the position loop of aerial inertially stabilized platform. Aerosp. Sci. Technol..

[2] Masten M.K. (2008). Inertially stabilized platforms for optical imaging systems. IEEE Control Syst. Mag..

[3] Zhao M. (2010). Locating of focal plane position for aerial cameras. Electron. Opt. Control.

[4] Liu J.H., Sun H., Zhang B., Dai M., Jia P., Shen H.H., Zhang L. (2007). Target self-determination orientation based on aerial photoelectric imaging platform. Opt. Precis. Eng..

[5] Li Y., Wang S.B., Ge W.Q. (2009). Precision analysis of opto-electric stable platform. J. Changchun Univ. Sci. Technol..

[6] Yuan H.W., Min Z.F., Zhang J.M. (2013). Design and Realization of a Three-Axis Stabilized Orthographic Platform for Aerial Survey. Electron. Opt. Control.

[7] Maleki L. (2011). Sources: The optoelectronic oscillator. Nat. Photonics.

[8] Ji S.P. (2018). Development and Key Technologies of Airborne Photoelectric Load Equipment. Aviat. Weapons.

[9] Xue D. (2011). Technical Development and Trend of Electro-optical Reconnaissance Platform. TRAINER.

[10] Li W.W., Guo Z.H., Chen J. (2015). Development status and trends of typical airborne optoelectronic sighting equipment in the US military. Aircr. Missiles.

[11] Li S.S., Zhong M.Y., Zhao Y. (2015). Estimation and compensation of unknown disturbance in three-axis gyro-stabilized camera mount. Trans. Inst. Meas. Control.

[12] Zhou X.Y., Gong G.H., Li J.P., Zhang H.Y., Yu R.X. (2015). Decoupling control for a three-axis inertially stabilized platform used for aerial remote sensing. Trans. Inst. Meas. Control.

[13] Li H.X., Gao Z.Y., Zhang R., Han F.T. (2007). Kinematic analysis of a four-gimbal space-stable platform and calculation of the motor torque. J. Tsinghua Univ..

[14] Naderolasli A., Tabatabaei M. (2017). Stabilization of the two-axis gimbal system based on an adaptive fractional-order sliding-mode controller. IETE J. Res..

[15] Shah S.V., Saha S.K., Dutt J.K. (2012). Denavit-Hartenberg parameterization of Euler angles. J. Comput. Nonlinear Dyn..

[16] Shah J.A., Rattan S.S., Nakra B.C. (2013). End-effector position analysis using forward kinematics for 5 DOF pravak robot arm. IAES Int. J. Robot. Autom..

[17] Zhang Z.J., Zhou H.B., Duan J.A. (2017). Design and analysis of a high acceleration rotary-linear voice coil motor. IEEE Trans. Magn..

[18] Xing L.G., Zhou H.X., Hou S.L., Cao R.M. (2011). Research and application of voice coil motor. Micromotors.

[19] Lee J., Wang W. (2012). Topological shape optimization of permanent magnet in voice coil motor using level set method. IEEE Trans. Magn..

[20] Dong W., Tang J. (2009). Design of a linear-motion dual-stage actuation system for precision control. Smart Mater. Struct..

[21] Dong W., Tang J., EIDeeb Y. (2015). Disturbance-observer-based control and related methods—An overview. IEEE Trans. Ind. Electron..

[22] Katsura S., Matsumoto Y., Ohnishi K. (2007). Modeling of force sensing and validation of disturbance observer for force control. IEEE Trans. Ind. Electron..

[23] Ginoya D., Shendge P.D., Phadke S.B. (2013). Sliding mode control for mismatched uncertain systems using an extended disturbance observer. IEEE Trans. Ind. Electron..

